# Arctic Diatoms as a Source of Antibiofilm Compounds: Identification of Methyl 3-Hydroxyoctadecanoate and Pheophorbide *a*

**DOI:** 10.3390/biom15101482

**Published:** 2025-10-21

**Authors:** Marit Huizer, Renate Osvik, Espen H. Hansen, Terje Vasskog, Jeanette H. Andersen, Kim van Wezel, Hans Christian Eilertsen, Johan Isaksson, Kine Ø. Hansen, Richard A. Ingebrigtsen

**Affiliations:** 1 Natural Products and Medicinal Chemistry Research Group, Department of Pharmacy, UiT—The Arctic University of Norway, NO-9037 Tromsø, Norway; 2 Marbio, Norwegian College of Fishery Science, UiT—The Arctic University of Norway, NO-9037 Tromsø, Norway; 3 Microalgae and Microbiomes, Norwegian College of Fishery Science, UiT—The Arctic University of Norway, NO-9037 Tromsø, Norway; 4 Chemical Synthesis and Analysis Group, Department of Chemistry, UiT—The Arctic University of Norway, NO-9037 Tromsø, Norway; 5 Cawthron Institute, Private Bag 2, Nelson 7042, New Zealand

**Keywords:** antibacterial activity, antibiofilm activity, arctic marine diatoms, bioprospecting, pheophorbide *a*, photobioreactor cultivation, methyl 3-hydroxyoctadecanoate, microalgae, secondary metabolites

## Abstract

Marine diatoms are prolific producers of bioactive metabolites, but Arctic species remain underexplored as sources of antibacterial and antibiofilm agents. Here, seven species were grown in photobioreactors (PBRs) and systematically screened for antibacterial, antibiofilm, and cytotoxic activities. All strains inhibited Gram-positive bacteria, and four reduced *Staphylococcus epidermidis* biofilm formation. *Porosira glacialis* emerged as a lead species, combining potent antibiofilm activity with favourable traits for large-scale cultivation, and no detectable cytotoxicity. Bioactivity-guided fractionation of *P. glacialis* yielded two antibiofilm compounds: methyl 3-hydroxyoctadecanoate, the first time reported in diatoms and newly associated with antibiofilm bioactivity, and pheophorbide *a*, a chlorophyll degradation product. Both inhibited *S. epidermidis* biofilm formation without any observed cytotoxicity. Notably, *Cylindrotheca closterium* exhibited cultivation-dependent antibiofilm activity, underscoring the importance of growth conditions for metabolite production. These findings highlight the potential of Arctic diatoms as a sustainable source of antibiofilm agents and support further exploration of their metabolites for antimicrobial and industrial applications.

## 1. Introduction

Microalgae are photosynthetic microorganisms capable of synthesising high-value biomolecules, including lipids, pigments, proteins, and bioactive compounds [1,2,3,4,5,6]. Among these, diatoms represent one of the most ecologically significant groups, contributing up to 40% of marine primary production and nearly 20% of global carbon fixation [7,8,9,10]. Diatoms produce a diverse range of metabolites, including polyunsaturated fatty acids (PUFAs), pigments, alkaloids, and terpenoids [11]. These compounds function as chemical defences to withstand environmental challenges, deter predation, and facilitate intercellular communication [12]. The types and concentrations of these metabolites are influenced by environmental conditions, geographical location, and seasonality, resulting in distinct chemical profiles with potentially diverse bioactivities [11,13,14,15,16,17]. Their ecological dominance is due to adaptive strategies that enable them to thrive in nutrient-poor and fluctuating conditions [11,12,18].

Arctic marine diatoms are adapted to extreme conditions, including low temperatures, extreme light fluctuations, and varying nutrient availability. These environmental pressures can shape the production of secondary metabolites, potentially leading to unique chemical structures and bioactivities. They also have been shown to enhance their production of n-3 PUFAs such as eicosapentaenoic acid (EPA) and docosahexaenoic acid (DHA), which are essential for human health but cannot be synthesised in the body [19,20,21,22,23,24].

Arctic diatoms synthesise a spectrum of bioactive compounds, including fucoxanthin, chlorophylls, and β-carotene [3,4,24,25]. These compounds exhibit antioxidant, anti-inflammatory, and antimicrobial properties, making them attractive for applications in nutraceuticals for disease prevention. For example, the Arctic diatom *Porosira glacialis* has demonstrated antibiofilm activity against *Staphylococcus epidermidis*, indicating potential to mitigate biofouling and enhance the stability of photobioreactor (PBR) systems [26,27]. Other cold-adapted species have also shown bioactive properties, including two Antarctic diatoms [28] and six Nordic-simulated diatom strains [29].

Despite these promising attributes, the bioactive potential of Arctic diatoms remains underexplored, especially in the context of large-scale cultivation. Existing studies predominantly focus on single strains, with limited comparative analysis of multiple Arctic species under controlled growth conditions [15,16,30,31,32,33]. Moreover, key factors such as antibacterial, antibiofilm, and cytotoxic properties have not been systematically evaluated across multiple Arctic strains. This knowledge gap also extends to photobioreactor-based cultivation, where challenges such as biofouling and contamination limit industrial scalability [34].

To address these gaps, we conducted a comparative bioactivity screen of seven Arctic marine diatom strains cultivated at scale in PBR systems. These include *Porosira glacialis*, a marine diatom known for its suitability in high-volume PBR cultivation [35,36], and *Cylindrotheca closterium*, which is frequently used as a model in biofilm formation and ecotoxicology studies [37]. We evaluated their antibacterial, antibiofilm, and cytotoxic properties to assess their potential as viable, sustainable resources for biotechnology, aquaculture, and antimicrobial development.

## 2. Materials and Methods

### 2.1. Sample Collection and Strain Isolation

**Figure 1:**
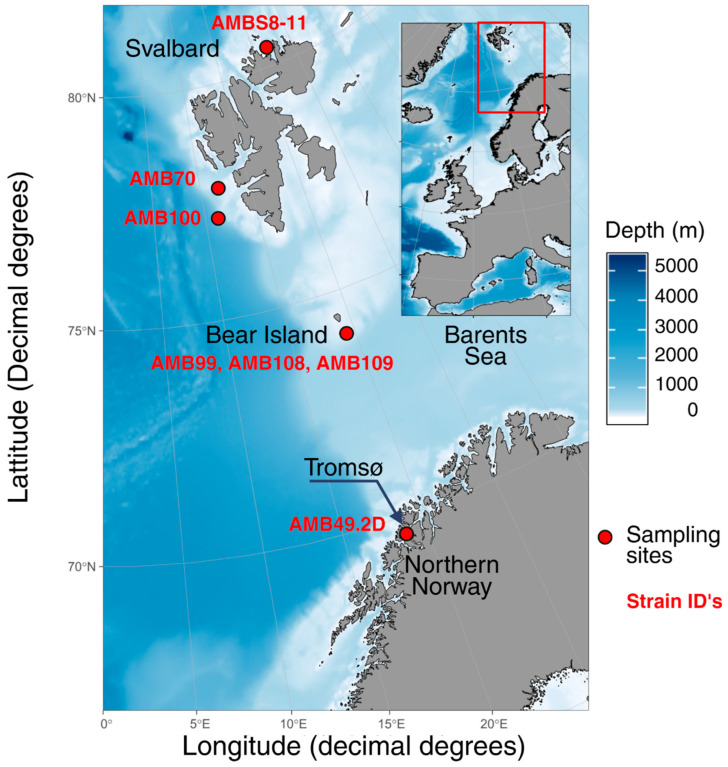
Sampling locations of Arctic diatom strains used in this study, generated using “gg0ceanMaps” [38]. Detailed shapefiles of Svalbard and the Norwegian coast in ggOceanMapsLargeData are from *Geonorge.no*.

Seawater and sediment samples were collected from waters close to Tromsø, Bear Island, and Svalbard during research cruises aboard the R/V Helmer Hanssen (Figure 1). Sediment was sampled using a Van Veen grab and stored at 4 °C in the dark until processing. Subsamples (~0.1 g) were inoculated in 30 mL sterile, pasteurised Guillard’s F/2 medium (Sigma-Aldrich, St. Louis, MO, USA) supplemented with silicate (1 mL/L, Na_2_O_3_Si·9H_2_O, Sigma-Aldrich, St. Louis, MO, USA) and incubated at 4 °C under a 14:10 light-dark cycle (80 µmol photons m^−2^ s^−1^) for ~14 days to stimulate germination. Seawater used for media preparation was filtered through a 0.22 μm membrane cartridge filter (Millipore, Burlington, MA, USA) to remove microorganisms.

Diatom strains were isolated from individual cells or chains under a stereomicroscope (100× magnification) using glass-blown, tapered Pasteur pipettes and maintained in a culture collection at UiT—The Arctic University of Norway (Table 1). Species identification was performed by morphological and molecular methods as described by Degerlund et al. [39].

### 2.2. Mass Cultivation in Photobioreactors

Seven Arctic diatom strains, each representing a distinct species, were cultivated in 630 L Plexiglas airlift-column PBRs under strain-specific light and temperature conditions (±1 °C, see Table 1). Cultivation was carried out in natural seawater enriched with Substral™ fertiliser (0.25 mL/L; Scotts Company (Nordics) A/S, Brøndby, Denmark) and silicate (1 mL/L, Na_2_O_3_Si·9H_2_O, Sigma-Aldrich, St. Louis, MO, USA). Cultures were illuminated on a 14:10 light-dark cycle and aerated with sterile compressed air. Growth and contamination were monitored microscopically (400× magnification) throughout the cultivation period. Biomass was harvested using sequential plankton net filtration (20 µm and 10 µm) and centrifugation (2500 rpm, 10 min), then flash-frozen in liquid nitrogen and stored at −23 °C.

*P. glacialis* was further scaled to 300,000 L in an outdoor fibreglass column PBR under continuous light and nutrient-replete conditions (as described in Eilertsen et al. [40]). Filtered seawater was enriched with Yara Kristalon Purple (Yara International ASA, Oslo, Norway) and silicate to reach 90–100 μM nitrate and 18–59 μM silicate, measured using Spectroquant^®^ “Nitrate test in seawater” kits (Merck, Darmstadt, Germany). Cultivation was carried out at 5.1–5.9 °C with white/blu. e LED lighting (36–50 μmol photons m^−2^s^−1^ at 0.7 m depth, Li-Cor 193S Aspherical sensor). Biomass was harvested using an Evodos 10 centrifuge (Evodos B.V., Raamsdonksveer, The Netherlands) and stored at −80 °C.

### 2.3. Biomass Extraction and Fractionation

Freeze-dried biomass was extracted with 80% methanol (MeOH, Merck & Co, USA) as described by Ingebrigtsen et al. [15]. Extracts were filtered using Whatman no. 3 filters (<6 µm, Whatman plc, Maidstone, UK), evaporated to dryness using a Laborota 4002 rotary evaporator (Heidolph, Schwabach, Germany), and stored at −23 °C.

For all strains, extracts were fractionated using 6.5 g Diaion^®^ HP20SS resin (Supelco, Bellefonte, PA, USA) into eight fractions (F1–F8) using increasing polarity solvent gradients. Dried fractions were reconstituted in 100% dimethyl sulfoxide (DMSO) (Merck, Darmstadt, Germany) and diluted to 1 mg/mL for screening.

The complete extraction and flash fractionation protocol is provided in Supplementary Section S3.

### 2.4. Bioactivity Assays

The antibacterial testing methods were conducted following the methodology by Ingebrigtsen et al. [15]. Antibacterial activity was tested against three Gram-positive (*Enterococcus faecalis*, ATCC 29212; *Staphylococcus aureus*, ATCC 25923; *Streptococcus* Gr. B, ATCC 12386) and two Gram-negative (*Escherichia coli*, ATCC 25922; *Pseudomonas aeruginosa*, ATCC 27853) strains obtained from the American Type Culture Collection (ATCC; LGC Standards, Teddington, UK) using 96-well plates at 250 μg/mL. Growth control contained 50 µL bacterial solution and 50 µL sterile water. The blank consisted of 50 µL sterile water and 50 µL enrichment media. Gentamicin (Aventis Pharma, Frankfurt, Germany) served as the positive control. Optical density 600 (OD_600_) was recorded (1420 Multilabel Counter VICTOR^3^, PerkinElmer, Waltham, MA, USA), and activity was defined as OD_600_ < 0.05.

Biofilm inhibition assays were performed using *S. epidermidis* (ATCC 35984) following Lauritano et al. [16]. Samples were tested at 50 μg/mL in triplicate. Controls included *S. haemolyticus* (negative), *S. epidermidis* (positive), MilliQ water (blank), and medium. Biofilm formation was assessed by visual inspection and OD_600_ measurement.

Biofilm eradication assays were performed on pre-formed *S. epidermidis* biofilms grown in tryptic soy broth (TSB) + 1% glucose. After 24 h incubation at 37 °C, plates were washed with PBS, fresh TSB and 50 μL of test fractions were added, and plates were incubated again for 24 h. Biofilms were fixed, stained (e.g., crystal violet), and measured at 600 nm.

Cytotoxicity was evaluated in A2058 human melanoma cells (ATCC CRL-11147) using an MTS assay [15]. Cells were seeded in 96-well plates, incubated for 24 h at 37 °C, and then treated with test fractions. Cytotoxic activity was defined as <50% cell survival, with 40–50% survival classified as weakly active.

All bioactivity assays were performed in duplicate or triplicate, and results are reported as mean values from independent measurements. Supplementary Section S2 describes detailed assay protocols, including bacterial strains, controls, and assay conditions, and Supplementary Table S1 summarises this.

### 2.5. Re-Fractionation and Compound Purification

Flash fractions with detected activity were refractionated into 40 new fractions using a preparative high-pressure liquid chromatography (prep-HPLC) system equipped with a 600 HPLC pump, an XTerra PrepMS C8 (10 µm, 10 × 250 mm, Waters, Milford, MA, USA) column, a 2996 photodiode assay UV-detector, a 3100-mass spectrometer, and a 2767 sample manager (Waters, Milford, MA, USA). The solvents used were A: MQ-H_2_O and B: Acetonitrile (ACN) both with 0.1% formic acid (FA). The flow rate was 6 mL/min, with a gradient from 30% to 70% B over 40 min, and fractions were collected every minute. Fractions were transferred to 96-well plates, dried under vacuum, freeze-dried, and stored at −20 °C until further use. Prior to bioactivity testing, fractions were dissolved in 100% DMSO (Merck, Darmstadt, Germany) and MQ-H_2_O.

### 2.6. Compound Purification

The initial purification of the compounds was performed using the same prep-HPLC-MS set-up as for the HPLC fractionation. Several gradients of A: MQ-H_2_O + 0.1% FA and B: ACN + 0.1% FA were used in the purification process to achieve the best possible separation and isolation of the selected compounds. The second purification step was performed using a semi-preparative HPLC system coupled to a PDA and QDa-MS. For separation, an Atlantis^TM^ T3 C18 column (3 µm, 3.0 × 150 mm, Waters, Milford, MA, USA) was used. Solvents were A: MQ-H_2_O + 0.1% FA and B: ACN + 0.1% FA. Gradients were adapted to achieve the best possible separation and isolation of the selected compounds.

The yields of purified compounds were calculated relative to the dry biomass extracted. From a 300,000 L *P. glacialis* culture, 500 g of wet biomass (≈50 g dry weight, DW) was processed, yielding 8.9 g of organic extract. A 1.5 g subsample of this extract was subjected to purification. Yields were expressed both as milligrams per gram of dry biomass (mg g^−1^ DW) and as a percentage of the extract weight (% *w*/*w*). Detailed yield calculations are provided in Supplementary Table S3.

### 2.7. Structural Elucidation

The selected compounds were analysed by high-resolution mass spectrometry (HRMS) to determine their molecular formula, followed by nuclear magnetic resonance (NMR) spectroscopy for detailed structural elucidation and confirmation in extracts of *P. glacialis*.

High-resolution mass spectrometry was performed using a Thermo Scientific IDX Tribrid Orbitrap mass spectrometer (Thermo Fisher Scientific, Waltham, MA, USA) equipped with an electrospray ionisation (ESI) source, operating in positive ionization mode. Full MS scans were acquired in the Orbitrap at a resolution of 120,000 over a *m*/*z* range of 250–1500. Precursor ions exceeding an intensity threshold of 1.0 × 10^5^ were isolated with a 1.5 *m*/*z* window and fragmented by stepped higher-energy collisional dissociation (HCD, 25%, 30%, 35%). MS^2^ spectra were recorded in the Orbitrap at a resolution of 15,000.

NMR spectra were acquired in *d*_6_-DMSO using a Bruker Avance III HD spectrometer (Bruker, Billerica, MA, USA) operating at 600 MHz for protons. The spectrometer was equipped with an inverse TCI cryoprobe enhanced for ^1^H, ^13^C, and ^2^H nuclei. The chemical shifts for ^1^H and ^13^C were referenced to the residual solvent peak (*d*_1_-DMSO: δ_H_ = 2.50 ppm, δ_C_ = 39.52 ppm).

### 2.8. Software

Microscopy images were captured using Leica DFC320 (Leica Microsystems, Wetzlar, Germany) and Leica Acquire software (v3.4.1). Images were assembled in Adobe Photoshop CC (v14.1.2) and finalised in Adobe Illustrator CC 2015. Data analyses were performed in R (v3.2.2), with visualisation using the ggplot2 and ggOceanMaps packages [38].

Supplementary Figure S1 provides a schematic overview of the experimental pipeline, showing the sequence from strain isolation to compound identification.

## 3. Results

### 3.1. Bioactivity Screening of Arctic Diatoms

**Figure 2:**
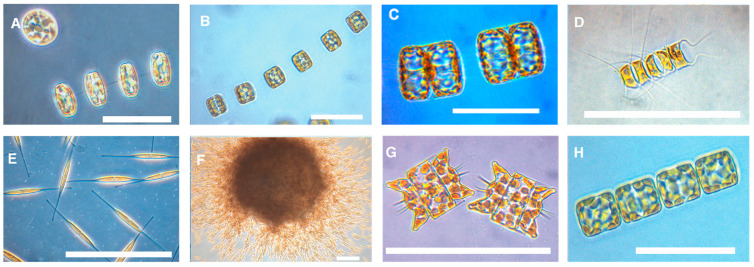
Live cell images of diatoms used in this study. (**A**) A chain of *T. hyalina* with one single cell in valve view. (**B**) A chain of vegetatively growing *T. antarctica* var. borealis cells. (**C**) Dividing cells of *P. glacialis*. (**D**) A chain of *C. karianus*. (**E**) Cells of *C. closterium*. (**F**) A dense, typically spherical colony of *C. closterium* observed during mass cultivation in airlift photobioreactors. (**G**) Cells of *O. aurita*. (**H**) A chain of *B. bathyomphala*. All scale bars = 100 µm. Photos by RAI.

**Figure 3:**
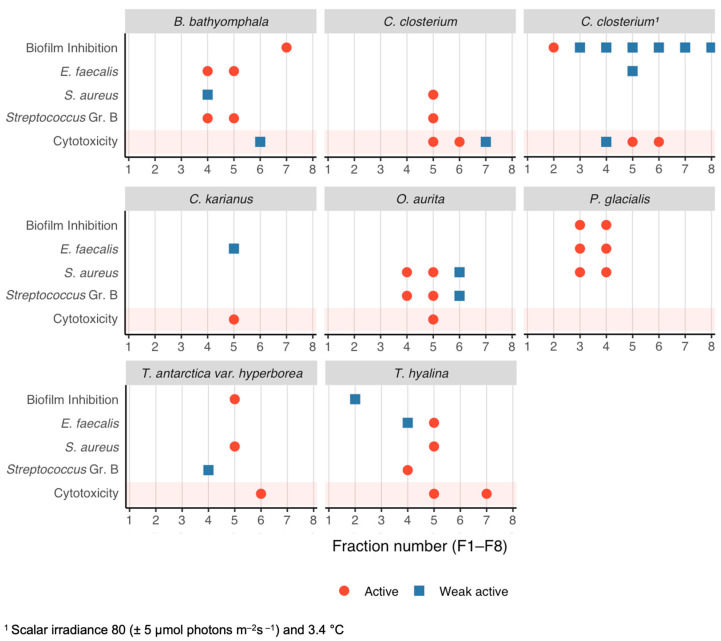
Bioactivity profiles of eight sequential fractions (F1–F8) from seven Arctic diatom strains. Each facet shows hits per assay row: biofilm inhibition, antibacterial activity against *Enterococcus faecalis*, *Staphylococcus aureus*, *Streptococcus* Gr. B and cytotoxicity. Red circles indicate active fractions; blue squares indicate weakly active fractions. Activity was classified as Active (A) (<0.05 OD_600_ or <50% cell survival), Weak (W) (40–50% survival or near-threshold OD_600_), or Inactive. The red band highlights the cytotoxicity read-out. Species (left to right, top to bottom): *B. bathyomphala*, *Cylindrotheca closterium* (two cultivation regimes), *Chaetoceros karianus*, *Odontella aurita*, *Porosira glacialis*, *Thalassiosira antarctica* var. *hyperborea*, and *T. hyalina*. All bioactivity assays were performed in triplicate.

The bioactive potential of the seven Arctic marine diatom strains (Figure 2) was evaluated through a multi-assay screening for antibacterial, antibiofilm, and cytotoxic effects. Each strain’s crude extract was fractionated (F1–F8), and all fractions were tested against three Gram-positive bacteria (*Streptococcus* Gr. B, *E. faecalis*, *S. aureus*), two Gram-negative bacteria (*E. coli*, *P. aeruginosa*), as well as for inhibition of *S. epidermidis* biofilm formation and for cytotoxicity on human cells. The activity was classified as Active (A), where OD_600_ measured < 0.05 for bacterial and biofilm inhibition and a cell survival of <50% in the cytotoxicity assay, Weakly Active (W), or Inactive, as summarised in Figure 3.

All seven diatom species showed some degree of bioactivity, particularly against Gram-positive bacteria. Notably, every strain yielded at least one fraction active against *Streptococcus* Gr. B, *E. faecalis*, or *S. aureus*. None of the fractions exhibited activity against the Gram-negative *E. coli* or *P. aeruginosa*. Four of the seven species also produced fractions that inhibited *S. epidermidis* biofilm formation (Figure 3). In terms of cytotoxicity, most fractions were non-cytotoxic, with only a few instances of weak cytotoxic effects at the highest test concentration.

Among the strains, *P. glacialis* demonstrated the most consistent and potent bioactivity across the assays. Fractions F3 and F4 from *P. glacialis* were active in inhibiting *S. epidermidis* biofilm formation and suppressed growth of *E. faecalis* and *S. aureus*, all without detectable cytotoxicity. *B. bathyomphala* also showed a broad activity profile, with an F7 fraction that inhibited biofilm formation and fractions active against *Streptococcus* and *E. faecalis*; however, one fraction (F6) from *B. bathyomphala* showed weak cytotoxicity. *T. hyalina* presented strong antibacterial activity (fractions active against all three Gram-positive bacteria), but several of its fractions (F5 and F7) were cytotoxic, which diminishes its appeal for drug discovery. *T. antarctica var. hyperborea* produced fractions (especially F5) with antibiofilm and antibacterial activity, but again an adjacent fraction (F6) was cytotoxic. In contrast, *C. closterium*, *O. aurita*, and *C. karianus* exhibited the lowest levels of bioactivity in this screen—most fractions from these species were inactive in both antibacterial and antibiofilm assays, and none were strongly cytotoxic either.

An interesting observation was made for *C. closterium*: significant biofilm inhibition was seen only in the fractions from the culture grown under low-temperature, low-light conditions (3.4 °C, 80 μmol photons m^−2^ s^−1^; Table 1). The same strain grown at a higher temperature and light (8 °C, 130 μmol photons m^−2^ s^−1^) did not yield any antibiofilm-active fractions. This cultivation-dependent difference suggests that environmental conditions can induce or suppress the production of bioactive metabolites in *C. closterium*, exemplifying a OSMAC (“One Strain, Multiple Compounds”) effect, where stressing the organism (here, by colder, darker conditions) leads to different chemistry.

### 3.2. Cultivation Performance and Selection of Lead Strain

Cultivation of the seven strains in airlift PBRs revealed strain-specific differences in growth and harvestability. *T. hyalina*, for example, formed long chains that tended to clog the outlet filters and pipelines, making harvest inefficient. *B. bathyomphala* grew well but exhibited high cell loss during filtration (the small cells passed through or broke apart), reducing overall biomass recovery. *T. antarctica var. hyperborea* achieved decent biomass and showed bioactivity, but its inherent cytotoxicity (Figure 3, as noted in several fractions) made it unsuitable for further development as an antimicrobial source. *C. karianus*, *C. closterium*, and *O. aurita* all grew, but their relatively weak bioactivity profiles (Figure 3) provided little incentive to prioritize them for upscaling.

*P. glacialis* distinguished itself by robust cultivation traits. This large centric diatom grew to high densities likely aided by its sizable cells which settled well and were easy to harvest via gentle centrifugation. Importantly, *P. glacialis* cultures showed no detectable cytotoxicity (Figure 3). Given this combination of favorable cultivation characteristics, broad antibiofilm and antibacterial activity, and lack of cytotoxicity, *P. glacialis* was selected as the lead strain for upscaling and a more detailed chemical investigation. And indeed, in a 300 m^3^ culture (Section 2.2), *P. glacialis* sustained healthy for several months as reported also by Eilertsen et al. [35,36].

### 3.3. Bioactivity-Guided Fractionation of P. glacialis

To isolate the specific metabolites responsible for *P. glacialis’* bioactivities, we performed bioactivity-guided fractionation on its extract. The active flash fractions F3 and F4 from *P. glacialis* (identified in the initial screen as inhibiting *S. epidermidis* biofilm and Gram-positive bacteria) were fractionated by preparative HPLC into 40 subfractions. Each subfraction was then tested for antibiofilm activity (inhibition of *S. epidermidis* biofilm formation and disruption of pre-formed biofilms) and for antibacterial activity against the same five bacteria.

**Figure 4:**
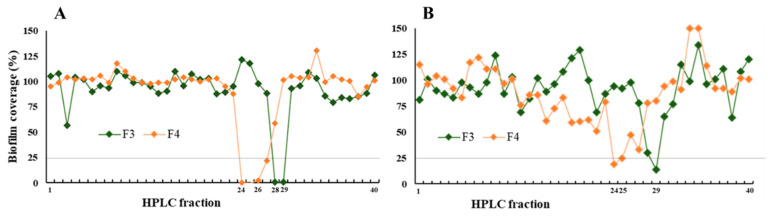
(**A**) Inhibition of biofilm formation and (**B**) removal of pre-formed biofilms by 40 HPLC fractions derived from flash fractions F3 and F4 of *P. glacialis* extracts. Biofilm coverage was measured after treatment, expressed as a percentage relative to untreated controls. Fractions showing less than 25% biofilm coverage (indicated by the horizontal line) were considered active. F3 (green) and F4 (orange) are shown separately to illustrate reproducibility and variation in activity profiles across both assays. Both assays were performed in duplicate.

Six subfractions inhibited *S. epidermidis* biofilm formation (Figure 4A). Three subfractions were also able to remove a considerable portion of established biofilms (Figure 4B). Importantly, none of the subfractions showed cytotoxicity consistent with the parent fractions.

**Figure 5:**
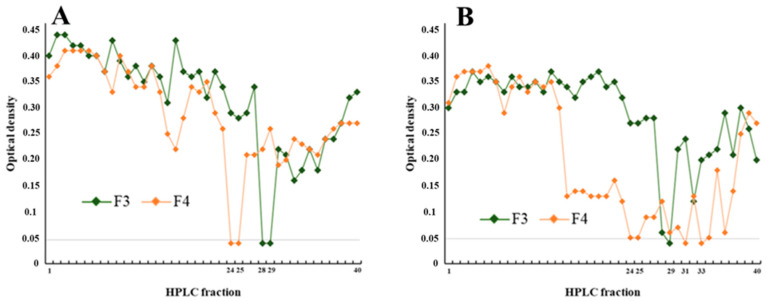
Growth inhibition of (**A**) *Staphylococcus aureus* and (**B**) *Enterococcus faecalis* by 40 HPLC fractions re-fractionated from flash fractions F3 (green) and F4 (orange) of *P. glacialis* extracts. Optical density (OD_600_) was measured after incubation, and fractions with OD < 0.05 (horizontal line) were considered active. The profiles illustrate distinct antibacterial responses across the fraction series, with several active hits detected in both bacterial assays. Both assays were performed in duplicate.

Four subfractions inhibited the growth of *S. aureus* and six inhibited *E. faecalis* (as shown in Figure 5A and Figure 5B, respectively). As expected, no activity was seen against *E. coli* or *P. aeruginosa* at this stage, reinforcing the earlier observation.

### 3.4. Compound Isolation and Screening of Active Compounds

Bioactive subfractions from *P. glacialis* yielded five compounds (A–E) in the following amounts: A (0.2 mg), B (0.9 mg), C (1.5 mg), D (1.5 mg), and E (3.7 mg), corresponding to 0.024–0.438 mg g^−1^ dry biomass. Detailed extraction and yield data, normalized to dry biomass, are provided in Supplementary Table S3.

**Figure 6:**
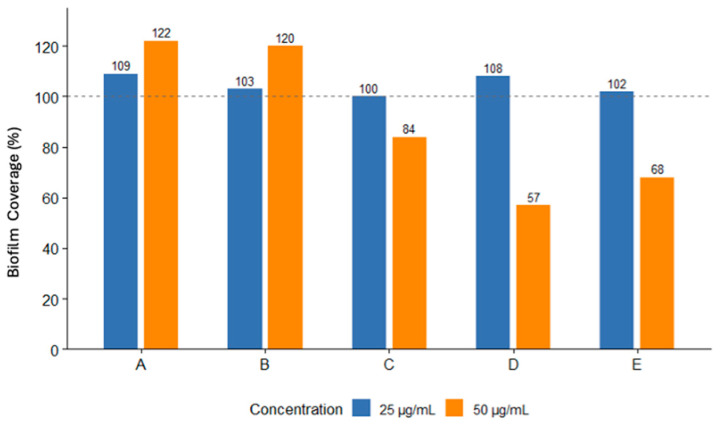
Bioactivity testing of isolated compounds A-E for inhibition of biofilm formation. Biofilm coverage was measured as a percentage of biofilm after 24 h. Assays were performed in duplicate.

Antibiofilm activity was evaluated at 50 µg/mL and 25 µg/mL of the purified compounds (Figure 6). Compounds D and E exhibited antibiofilm activity at 50 µg/mL, reducing biofilm formation by 57% and 68%, respectively. At the lower concentration (25 µg/mL), both compounds showed weaker effects, with biofilm levels approaching or exceeding those of untreated controls.

Compounds A–C were inactive in this assay, with no reduction in biofilm formation at either concentration. Instead, they appeared to increase biofilm formation, resulting in up to 120% biofilm coverage. None of the isolated compounds showed significant activity in bacterial growth inhibition or cytotoxicity assays.

### 3.5. Structural Elucidation

**Figure 7:**
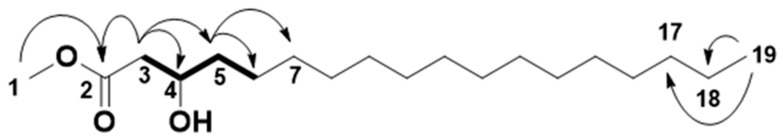
NMR-Based structural elucidation of Compound D with Selected HMBC (arrow) and COSY (bold) correlations.

The molecular structures of Compounds D and E were elucidated using HRMS and further verified by NMR spectroscopy where necessary (Table 2, Figure 7).

Compound D exhibited a protonated molecular ion [M+H]^+^ at *m*/*z* 315.2895 and a sodium adduct [M+Na]^+^ at *m*/*z* 337.2715. The elemental composition of the protonated species was determined as C_19_H_39_O_3_^+^ (*m*/*z* 315.2899, mass error: 1.27 ppm), corresponding to the neutral molecular formula C_19_H_38_O_3_ (monoisotopic mass 314.2821 Da). The MS/MS spectrum showed a prominent fragment ion at *m*/*z* 297.1855, consistent with the neutral loss of water (–H_2_O), indicative of a hydroxylated species. Further fragments were observed at *m*/*z* 283.1069 and 255.1128, arising from alkyl chain cleavages. However, these fragments did not allow an unambiguous assignment of the hydroxyl group’s position or type.

To confirm the structure, additional structural confirmation was carried out using 1D (^1^H, ^13^C) and 2D (HSQC, HMBC, COSY) NMR. While overlap among aliphatic CH_2_ signals (C8–C16) limited full assignment, COSY and HMBC correlations confirmed positions C1–C7 and C17–C19 ( Figure 7 and Figures S2–S4, Table S4). The proton at δH 3.80 (δC 67.1) was identified as a hydroxyl-bearing CH group. It showed cross-peaks to adjacent methylene groups at δH 2.39 and 2.28 (δC 42.4), consistent with the presence of a secondary hydroxyl at C3. These correlations also confirmed the connectivity between the ester methyl group (C1), the α- and β-carbons (C2–C3), and the remainder of the saturated chain. Combined with the HRMS data, these results provide conclusive support for the identification of Compound D as methyl 3-hydroxyoctadecanoate (Figure 7).

Compound E presented a protonated molecular ion at *m*/*z* 593.2765, along with a sodium adduct at *m*/*z* 615.2578. Its elemental composition, C_35_H_37_N_4_O_5_^+^ (*m*/*z* 593.2764; mass error 0.17 ppm) matched pheophorbide *a*, a known chlorophyll degradation product. Fragmentation produced characteristic ions at *m*/*z* 547.2698 (−COOH), *m*/*z* 533.2545 (−CH_3_), *m*/*z* 575.2652 (−H_2_O), and *m*/*z* 461.2328. As this compound is well-documented in diatoms, no further structural verification was deemed necessary.

NMR assignments and mass spectra for bioactive compounds are provided in Supplementary Sections S5 and S6, respectively.

## 4. Discussion

This study demonstrates that Arctic marine diatoms are a promising source of antimicrobial agents. All seven tested Arctic diatom strains exhibited activity against Gram-positive bacteria, and four significantly inhibited *S. epidermidis* biofilm formation. *P. glacialis* emerged as the lead strain, combining antibiofilm activity with advantageous traits for mass cultivation and no detectable cytotoxicity.

### 4.1. Bioactivity in Arctic Diatoms

The Arctic diatoms in our study exhibited bioactivity even under nutrient-replete, non-stressed conditions. This contrasts with observations in temperate microalgae: for example, Lauritano et al. [16] reported that two *Leptocylindrus* species required nitrogen starvation to trigger antibiofilm compounds, achieving up to ~90% inhibition of *S. epidermidis* biofilms only under nutrient stress. In their broad screening, antibacterial effects were generally weak, with *Skeletonema marinoi* showing activity against *S. aureus* only in certain conditions [16]. The Arctic strains, by comparison, produced antibacterial and antibiofilm metabolites constitutively, suggesting that cold-adapted diatoms may maintain elevated baseline levels of chemical defenses. The consistent bioactivity observed in our Arctic isolates aligns with this idea of chemical preadaptation, conferring a selective advantage in competitive ecosystems.

At the same time, our results highlight the profound influence of cultivation conditions on diatom bioactivity, supporting the “One Strain, Many Compounds” (OSMAC) strategy [16]. *C. closterium* showed antibiofilm activity only when grown under low temperature and low light, whereas cultures at higher temperature and irradiance were inactive. This condition-dependent activity suggests that certain bioactive metabolites in *C. closterium* are upregulated by environmental cues (in this case, simulating polar light and temperature). Our findings indicate that the biofilm-inhibiting potential of *C. closterium* can be “switched off” by suboptimal culture conditions, which is consistent with other reports that elevated temperature or light can diminish diatom bioactivity [11,15,16]. Diatoms such as *Nitzschia* cf. *pellucida* likewise adjust their chemical defences in response to external stimuli, highlighting the role of ecological pressures in shaping metabolite production [41].

This underscores the importance of standardizing or carefully reporting culture conditions in bioactivity assays. *C. closterium* is frequently used as a model microalga in biofouling assays [42] and from an OSMAC perspective, altering growth parameters (light intensity, temperature, nutrient levels, or other stresses) can induce differential expression of secondary metabolites [43,44]. This should be considered when interpreting assay results or comparing data across studies. *C. closterium*’s status as a cosmopolitan species has been questioned [45], and close examination of similar instances of diatom cosmopolitanism has unveiled cryptic diversity [39,46], which could in turn give rise to inconsistencies [47].

Finally, the consistent activity observed in the Arctic strains likely reflects evolutionary adaptations to cold, nutrient-poor marine environments. In such ecosystems, intense microbial competition for limited nutrients likely drives the constitutive production of chemical defenses (e.g., antimicrobial lipids and pigments) as a selective advantage. Collectively, our findings expand the ecological relevance of diatom-derived metabolites and suggest that adaptation to polar conditions is an important driver of secondary metabolite biosynthesis.

To further clarify how cultivation parameters influence diatom bioactivity, future large-scale studies could integrate untargeted metabolomics and lipidomics to identify how metabolite and lipid profiles vary under different light and temperature regimes. Such approaches would provide a broader understanding of condition-dependent bioactivity and compound production in Arctic diatoms.

### 4.2. Antibiofilm Compounds Isolated from P. glacialis

The biofilm assays in this study focused on *S. epidermidis*, a well-established model for biofilm formation and a relevant strain frequently used in antibiofilm research [16]. Testing against other biofilm formers such as *Pseudomonas aeruginosa*, *Escherichia coli*, or mixed-species communities would provide valuable comparative insights into spectrum and selectivity of activity.

The bioactivity-guided fractionation of *P. glacialis* yielded two compounds responsible for its antibiofilm effect. The first active metabolite was identified as methyl 3-hydroxyoctadecanoate (Compound D), a fatty acid methyl ester (FAME). To our knowledge, this is the first report of methyl 3-hydroxyoctadecanoate in a diatom, and the first time it has been linked to antibiofilm or antimicrobial activity. This compound was previously detected in unrelated organisms (e.g., higher plants and bacteria) but not associated with bioactivity (*Minuartia recurva* and *Acokanthera schimperi* [48,49], *Thiobacillus thioxidans* [50], *Melicocca bijuga* [51]). The discovery of an antibiofilm FAME in *P. glacialis* is noteworthy given the well-recognized antimicrobial properties of free fatty acids and their derivatives [52]. Even trace amounts of certain fatty acids can inhibit microbial growth [52], and many microalgae (including diatoms) produce polyunsaturated fatty acids like eicosapentaenoic acid (EPA) known to suppress various bacteria, including methicillin-resistant *S. aureus* (MRSA) [53,54,55,56,57]. Other free fatty acids are known to inhibit the growth or development of bacteria [43], fungi [57,58,59,60,61,62,63], cyanobacteria [64,65], protozoa [66] and viruses [67]. Recent studies have identified several bioactive lipid classes in microalgae, such as monogalactosyldiacylglycerols (MGDG), sulfoquinovosyldiacylglycerols (SQDG), and lysophosphatidylcholines (LPC), all of which exhibit antibiofilm effects [68]. In general, hydrophobicity and chain length of fatty acid-derived molecules are important factors for penetrating biofilm matrices and disrupting microbial adhesion [69]. Methyl 3-hydroxyoctadecanoate fits within this bioactive lipid paradigm, likely acting by integrating into bacterial cell membranes or biofilm exopolymeric substances, thereby impairing biofilm stability [70].

Other FAME components are commonly found in microalgae, as these compounds have been extensively studied for their potential as biodiesel [71,72,73]. Moreover, other FAMEs have demonstrated antibiofilm, antivirulence, and antibacterial properties, as seen in studies involving the coral-associated bacterium *Pseudomonas aeruginosa* [74], and various oral pathogens, including *Streptococcus mutans*, *Candida albicans*, and *Porphyromonas gingivalis* [21].

The second antibiofilm compound isolated was, pheophorbide *a* (Compound E), is a known chlorophyll degradation product [75]. Pheophorbide *a* reduced *S. epidermidis* biofilm formation by approximately 32% at 50 µg/mL in our tests, a moderate inhibition compared to the FAME but clearly above inactive compounds. Pheophorbide *a* and related chlorophyll derivatives are known bioactive pigments, with documented anticancer, antioxidant, and antimicrobial properties [31,75,76,77,78]. Trentin et al. recently found pheophorbide *a* in an Antarctic diatom and noted its strong antioxidant activity [28] suggesting such pigment derivatives contribute to the stress tolerance of microalgae. Other algal pigments have likewise been implicated in antimicrobial effects; for instance, the carotenoid lutein from *Chlorella* was shown to inhibit *Pseudomonas aeruginosa* biofilms [79]. The presence of pheophorbide *a* as an active principle in *P. glacialis* indicates that pigment degradation products can play a role in diatom chemical defense. While its antibiofilm efficacy was modest, it may act synergistically with other metabolites in the crude extract or be more potent against other microbial targets.

The precise mechanisms underlying the antibiofilm effects of the identified compounds remain to be clarified. Future studies should examine whether these metabolites influence bacterial adhesion, extracellular matrix production, or quorum-sensing pathways using microscopic, biochemical, or transcriptomic approaches.

### 4.3. Implications for Biofouling and Cultivation

The detection of antibiofilm compounds in Arctic diatoms has implications for both antifouling strategies and the cultivation of microalgae at scale. From a biotechnological standpoint, the ability of *P. glacialis* and other Arctic diatoms to generate antibiofilm compounds offers a natural solution to biofouling and bacterial infections. Biofouling is a pervasive challenge in aquaculture, shipping, water treatment, and algal cultivation systems, where bacterial biofilms or algal contaminants can clog equipment and outcompete beneficial organisms [80]. Currently, antifouling in aquaculture and water systems relies heavily on toxic biocides or copper-based materials, and no natural compounds are commercially available.

From an industrial biotechnology perspective, the ability to grow *P. glacialis* at scale and extract bioactive compounds opens opportunities for a sustainable supply chain of marine bioproducts. Unlike many marine invertebrates or deep-sea organisms that produce interesting metabolites but are hard to farm, microalgae can be cultured using simple inputs (light, seawater, CO_2_, and nutrients). The Arctic strain’s cold-adapted nature also means cultivation can occur in cold climates or seasons with lower risk of overheating and with possibly less contamination pressure from mesophilic microbes.

Despite growing interest in microalgal bioactivity, research on antibiofilm compounds from diatoms remains limited. This study contributes to the evidence that diatoms’ fatty acid and pigment profiles may shape their ecological interactions and biotechnological potential.

## 5. Conclusions

Through a comparative PBR-scale screening of seven Arctic diatom strains cultivated under controlled conditions, we demonstrate that Arctic diatoms consistently produce antibacterial and antibiofilm compounds, even in nutrient-replete environments. From an applied perspective, *P. glacialis* emerges as a promising lead strain: it combines robust growth and harvestability in large-scale photobioreactors with consistent antibiofilm and antibacterial activity and no detectable cytotoxicity. The results (i) establish that multiple Arctic diatoms inhibit *S. epidermidis* biofilm formation and selectively inhibit Gram-positive bacteria; (ii) identify methyl 3-hydroxyoctadecanoate and pheophorbide *a* as antibiofilm leads that act without detectable cytotoxic effects at screening concentrations; and (iii) validate *P. glacialis* as a scalable source of antibiofilm compounds.

Beyond *P. glacialis*, the cultivation-dependent activity observed in *C. closterium* underscores the importance of the OSMAC (“One Strain, Many Compounds”) principle in diatom bioactivity. Fractions from low-light, low-temperature cultures inhibited biofilm formation, whereas high-light, higher-temperature cultures were inactive. This highlights the role of environmental cues in modulating metabolite production and suggests that optimized culture regimes can be used to unlock hidden chemical diversity in diatoms.

Future work should focus on clarifying the mode of action of methyl 3-hydroxyoctadecanoate, exploring potential synergistic interactions with other metabolites. Together, these results expand our understanding of Arctic diatom chemistry and reinforce their potential as a sustainable source of novel antibiofilm agents.

## Figures and Tables

**Table 1:** Overview of Arctic diatom strains, identification methods, and cultivation conditions (irradiance and temperature) used in 630 L photobioreactor cultivations. *Porosira glacialis* was later scaled up to 300,000 L for bioassays and structural elucidation.

Strain ID	Species Name	Identification	Scalar Irradiance (μmol photons m^−2^ s^−1^) ^3^	Temperature (°C) ^4^	Origin
AMB99	*Bacteriosira bathyomphala*	LM ^1^	80	8	Sediment, Bear Island
AMB108	*Chaetoceros karianus*	LM	130	8.2	Sediment, Bear Island
AMBS8-11	*Cylindrotheca closterium*	LM	80	3.4	Sediment, Rijpfjorden, North Svalbard
		TEM ^2^	130	8	
AMB70	*Odontella aurita*	LM	130	8	Sediment, West Svalbard
AMB100	*Thalassiosira hyalina*	LM	130	8.1	Sediment, West Svalbard
AMB109	*T. antarctica var. hyperborea*	LM	130	9	Sediment, Bear Island
AMB49.2D	*Porosira glacialis*	LM	36–50	5.5	Water sample, Tromsøysund

^1^ Light microscopy. ^2^ Transmission electron microscopy ^3^ ±5 μmol photons m^−2^ s^−1^, ^4^ ±1 °C.

**Table 2:** HRMS data for compounds D and E isolated from *P. glacialis*.

Feature	Compound D	Compound E
Observed [M+H]^+^ (*m*/*z*)	315.2895	593.2765
Calculated [M+H]^+^ (*m*/*z*)	315.2899 (C_19_H_39_O_3_^+^)	593.2764 (C_35_H_37_O_5_N_4_^+^)
Mass error (ppm)	1.2687	0.1686
Neutral molecular formula	C_19_H_38_O_3_	C_35_H_36_O_5_N_4_
Neutral monoisotopic mass	314.2821 Da	592.2686 Da
Key fragment ions (*m*/*z*)	297.1855	533.2545
	283.1069	461.2328
	255.1128	575.2652
		547.2698
Identification	Methyl 3-hydroxyoctadecanoate	Pheophorbide *a*

## Data Availability

The original contributions presented in this study are included in the article and Supplementary Material. Further inquiries can be directed to the corresponding author(s), and the raw data supporting the conclusions of this article will be made available by the authors on request.

## Supplementary Materials

The following supporting information can be downloaded at: https://www.mdpi.com/article/10.3390/biom15101482/s1, Figure S1: Schematic Overview of Methodology; Table S1: Summary of controls used in bioactivity assays; Table S2: Flash Chromatography Gradient Scheme for Fractionation of Diatom Extracts; Table S3: Yields of isolated compounds from *P. glacialis*, expressed as percentage of extract and normalized to dry biomass; Table S4: ^1^H and ^13^C NMR assignments for compound D; Figure S2. ^1^H NMR spectrum of Compound D; Figure S3. 13C NMR spectrum of Compound D; Figure S4. HSQC + HMBC spectrum of Compound D; Figure S5: High-resolution mass spectrum of Compound D; Figure S6: High-resolution mass spectrum of Compound E.
