# Supplementary material for: Arctic Diatoms as a Source of Antibiofilm Compounds: Identification of Methyl 3-Hydroxyoctadecanoate and Pheophorbide a
Source: Biomolecules. 2025 Oct 21;15(10):1482. doi: 10.3390/biom15101482 (PMC12562383; doi:10.3390/biom15101482)
Supplement: Supplementary file 1 [file biomolecules-15-01482-s001.zip › biomolecules-3904240-supplementary.pdf]

Supplementary Information:

Arctic Diatoms as a Source of Antibiofilm Compounds: Identification of Methyl 3-Hydroxyoctadecanoate and Pheophorbide *a*

Marit Huizer [1,*], Renate Osvik [2], Espen H. Hansen [2], Terje Vasskog [1], Jeanette H. Andersen [2], Kim van Wezel [2], Hans Christian Eilertsen [3], Johan Isaksson [1,4], Kine Ø. Hansen [1] and Richard A. Ingebrigtsen [3,5]

[1] Natural Products and Medicinal Chemistry Research Group, Department of Pharmacy, UiT—the Arctic University of Norway, NO-9037 Tromsø, Norway; terje.vasskog@uit.no (T.V.); johan.isaksson@uit.no (J.I.); kine.o.hanssen@uit.no (K.Ø.H.)
[2] Marbio, Norwegian College of Fishery Science, UiT—The Arctic University of Norway, NO-9037 Tromsø, Norway; renate.osvik@gmail.com (R.O.); espen.hansen@uit.no (E.H.H.); jeanette.andersen@uit.no (J.H.A.); kim.v.wezel@uit.no (K.v.W.)
[3] Microalgae and Microbiomes, Norwegian College of Fishery Science, UiT—The Arctic University of Norway, NO-9037 Tromsø, Norway; hans.c.eilertsen@uit.no (H.C.E.); richard.ingebrigtsen@gmail.com (R.A.I.)
[4] Chemical Synthesis and Analysis Group, Department of Chemistry, UiT—The Arctic University of Norway, NO-9037 Tromsø, Norway
[5] Cawthron Institute, Private Bag 2, Nelson 7042, New Zealand
* Correspondence: marit.huizer@uit.no

Contents

S1. Schematical Overview of Methodology. 2
S2. Detailed Assay Protocols 3
Growth inhibition assay 3
Inhibition of biofilm formation 3
Eradication of established biofilm 4
Viability assay 4
S3. Extraction and Fractionation Procedures 6
S4. NMR Assignments 9
S5. Mass Spectrometry Data 12

S1. Schematic Overview of Methodology.

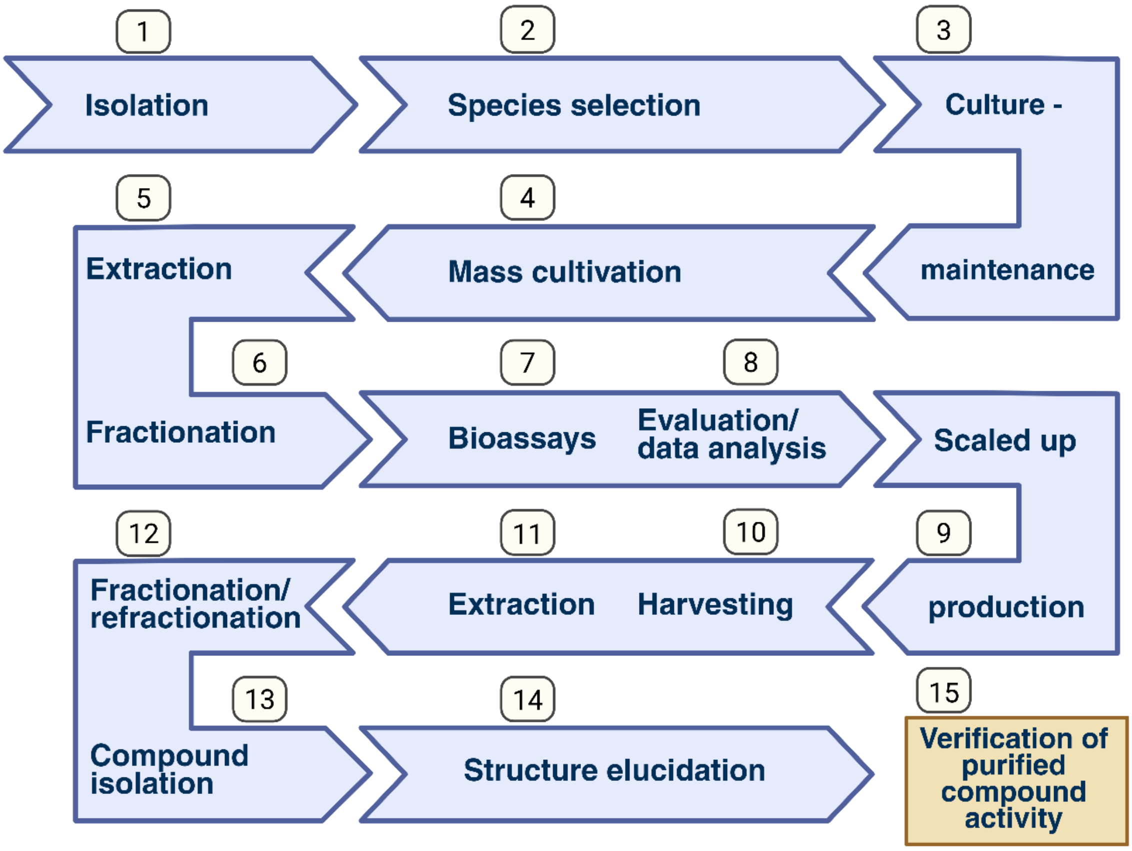

Figure S1: Flowchart of methodology used in the present study (*Created in https://BioRender.com).*

S2. Detailed Assay Protocols

Growth inhibition assay

In brief, the antibacterial assays used three strains of gram-positive (G+) bacteria and two strains of gram-negative (G-) bacteria obtained from the American Type Culture Collection Strains (ATCC, Manassas, VA, USA). The specific strain numbers are as follows: *Enterococcus faecalis* (G+, ATCC 29212), *Staphylococcus aureus* (G+, ATCC 25923), *Streptococcus agalactiae* (*Streptococcus* Gr. B, G+, ATCC 12386), *Escherichia coli* (G-, ATCC 25922) and *Pseudomonas aeruginosa* (G-, ATCC 27853). The flash fractions were tested at a concentration of 250 µg/mL. Growth control samples consisted of 50 µL of bacterial solution mixed with 50 µL of sterile water. Negative controls were prepared by combining 50 µL of sterile water with 50 µL of enrichment media. Gentamicin (Aventis Pharma, Mumbai, India) was used as a positive control. The threshold for antibacterial activity was OD600 values below 0.05, as measured using a 1420 Multilabel Counter VICTOR^3 (PerkinElmer, Waltham, MA, USA). A visual inspection of the plates was also performed.

Inhibition of biofilm formation

The evaluation of the fractions' capability to inhibit biofilm formation by *Staphylococcus epidermidis* was conducted in accordance with the methodology described by Lauritano et al. (Lauritano et al., 2016). In brief, triplicate samples of flash fractions from diatom extracts were tested against the biofilm-forming bacterium *S. epidermidis* (ATCC 35984) at a final concentration of 50 µg/mL. The positive control was *S. epidermidis* cultured with MilliQ-water, while *Staphylococcus haemolyticus* was used as the negative control. The blank was prepared by combining MilliQ-water and enrichment media in a 1:1 ratio. To assess the impact on biofilm formation, the plates were visually inspected. Additionally, absorbance at 600 nm was measured using a 1420 VICTOR^3 Multilabel Counter (PerkinElmer).

Eradication of established biofilm

For the biofilm eradication assay, *S. epidermidis* was incubated overnight at 37 °C and subsequently diluted at a 1:100 ratio in tryptic soy broth (TBS) containing 1% glucose. A 96-well plate (Nunc) was prepared by adding 50 µL of the bacterial culture and 50 µL of deionized water (dH_2O) to each well. Blank samples were prepared by mixing TBS with 1% glucose and dH_2O in a 1:1 proportion. Negative control samples employed *S. haemolyticus*, while positive control samples used *S. epidermidi*s. The plate was then incubated at 37 °C for 24 h. After incubation, the plate was emptied and subjected to two washing steps with phosphate-buffered saline (PBS). Fresh TBS with 1% glucose was added to each well, along with test fractions (50 µL) in triplicate. Subsequently, the plate was incubated again at 37 °C for 24 h. Following the incubation, the plates were fixed, coloured, and measured using the same methodology applied in the biofilm formation inhibition assay.

Viability assay

The flash fractions were tested against the human melanoma A2058 cell line (ATCC CRL – 11147™) to assess potential cytotoxicity, using the colorimetric 3-(4,5-dimethylthiazol-2-yl)-5-(3-carboxymethoxyphenyl)-2-(4-sulfophenyl)-2H-tetrazolium (MTS) cell proliferation assay as described by Ingebrigtsen et al. (Ingebrigtsen et al., 2016). In brief, cells were seeded onto transparent Nunc Microwell 96-well microtiter plates (Thermo Fisher Scientific, MA, USA) and incubated at 37 °C for 24 h. The activity threshold was set at 50% survival, with survival rates ranging from 20% to 50% being categorized as weakly active.

Table S1: Summary of controls used in bioactivity assays.

**Assay Type**	**Control Type**	**Description**
Antibacterial Assay	Positive Control	Gentamicin (Aventis Pharma, India)
	Negative Control	50 µL sterile water + 50 µL enrichment media
	Growth Control	50 µL bacterial solution* + 50 µL sterile water

Biofilm Inhibition	Positive Control	*S. epidermidis* (ATCC 35984) + MilliQ water
	Negative Control	*S. haemolyticus*
	Blank	MilliQ water + enrichment media (1:1)
Biofilm Eradication	Positive Control	*S. epidermidis*
	Negative Control	*S. haemolyticus*
	Blank	TSB + 1% glucose + dH_2O (1:1)

**Bacterial strains: E. faecalis*, *S. aureus*, *S. agalactiae*, *E. coli*, *P. aeruginosa*

S3. Extraction and Fractionation Procedures

Fractionation was done using liquid-liquid extractions and flash fractionation to reduce chemical complexity. Aliquots of each extract (2 g) were dissolved in 30 mL 90% MeOH (Merck & Co.) and extracted with 60 mL hexane (Merck & Co.) using a separating funnel. MeOH phases were collected, and the hexane phase was re-extracted with 30 mL 90% MeOH. The combined MeOH phases were reduced to 1 mL using a Laborota 4002 rotavapor (Heidolph) and then transferred to a test tube. 2 g of Diaion® HP20SS resin (Supelco Analytical, Charlottesville, NA, USA) was added, followed by complete drying under vacuum using a Heraeus Multifuge 3S-R centrifugal evaporator (Kendro, Ostende, Germany). The subsamples were separated into eight fractions using a Biotage™ SP4 Flash Chromatography system (Charlotte, NC).

The sample was introduced at the top of self-packed Biotage SNAP columns (Uppsala, Sweden) containing 6.5 g of Diaion® HP20SS resin (Supelco Analytical). The solvent gradient used for flash chromatography is detailed in Table S2.

Table S2: Flash Chromatography Gradient Scheme for Fractionation of Diatom Extracts.

Step	Solvent Composition	Duration	Fraction Collected
1	5:95% MeOH:H2O	6 min	F1
2	25:75% MeOH:H2O	6 min	F2
3	50:50% MeOH:H_2O	6 min	F3
4	75:25% MeOH:H_2O	6 min	F4
5	100% MeOH	12 min	F5
6	50% MeOH 50% Acetone	6 min	F6
7	100% Acetone	12 min	F7 (6 min) F8 (6 min)

All fractions were dried using a Laborota 4002 rotavapor (Heidolph), weighed, and then dissolved in 100% dimethyl sulfoxide (DMSO, Merck & Co.) at a concentration of 25 µL/mg sample.

Mass-cultivated samples of *P. glacialis* were freeze-dried prior to extraction for MS analysis. Extraction was performed using a mixture of dichloromethane (DCM) and methanol (DCM: MeOH, 1:1 v/v) at a ratio of 20 mL per gram of residue at 4 °C for 20 h. The supernatant was collected in a round-bottom flask and re-extracted with DCM: MeOH (1:1 v/v) at 4 °C for 1 h. The combined supernatants were dried using a rotavapor, yielding a dehydrated organic extract. The samples were dissolved in isopropanol to a concentration of 1 mg/mL before analysis.

S4. Compound Yields and Normalization

Table S3 shows the yields of purified compounds from *Porosira glacialis*, expressed as milligrams per gram dry biomass (mg g^{-1} DW) and as a percentage of extract mass (% w/w). Calculations were based on 1.5 g extract derived from 50 g dry biomass (corresponding to 500 g wet biomass) obtained from the 300,000 L cultivation.

Table S3: Yields of isolated compounds from P. glacialis, expressed as percentage of extract and normalized to dry biomass.

Compound	Amount Isolated (mg)	% of extract (w/w)	mg g^{-1} dry biomass
A	0.2	0.013 %	0.024
B	0.9	0.060 %	0.107
C	1.5	0.100 %	0.178
D	1.5	0.100 %	0.178
E	3.7	0.247 %	0.438

S5. NMR Assignments

Table S4: ^{1}H and ^{13}C NMR assignments for compound D.

	δ_C, type	δ_H, type (*J* in Hz)
1	51.1, CH_3	3.57, s
2	171.8, C	
3a	42.4, CH_2	2.39, dd (14.7, 4.7)
3b		2.28, dd (14.7, 8.3)
4	67.1, CH	3.80, m
5	36.9, CH_2	1.39 - 1.30, m*
6a	25.0, CH_2	1.39 - 1.30, m*
6b		1.24, m*
7	28.7, CH_2	1.28 - 1.18, m*
8	29.1 - 29.0, CH_2*	1.28 - 1.18, m*
9	29.1 - 29.0, CH_2*	1.28 - 1.18, m*
10	29.1 - 29.0, CH_2*	1.28 - 1.18, m*
11	29.1 - 29.0, CH_2*	1.28 - 1.18, m*
12	29.1 - 29.0, CH_2*	1.28 - 1.18, m*
13	29.1 - 29.0, CH_2*	1.28 - 1.18, m*
14	29.1 - 29.0, CH_2*	1.28 - 1.18, m*
15	29.1 - 29.0, CH_2*	1.28 - 1.18, m*
16	29.1 - 29.0, CH_2*	1.28 - 1.18, m*
17	31.3, CH_2	1.23, m*
18	22.1, CH_2	1.25, m*
19	14.0, CH_3	0.85, t (6.9)

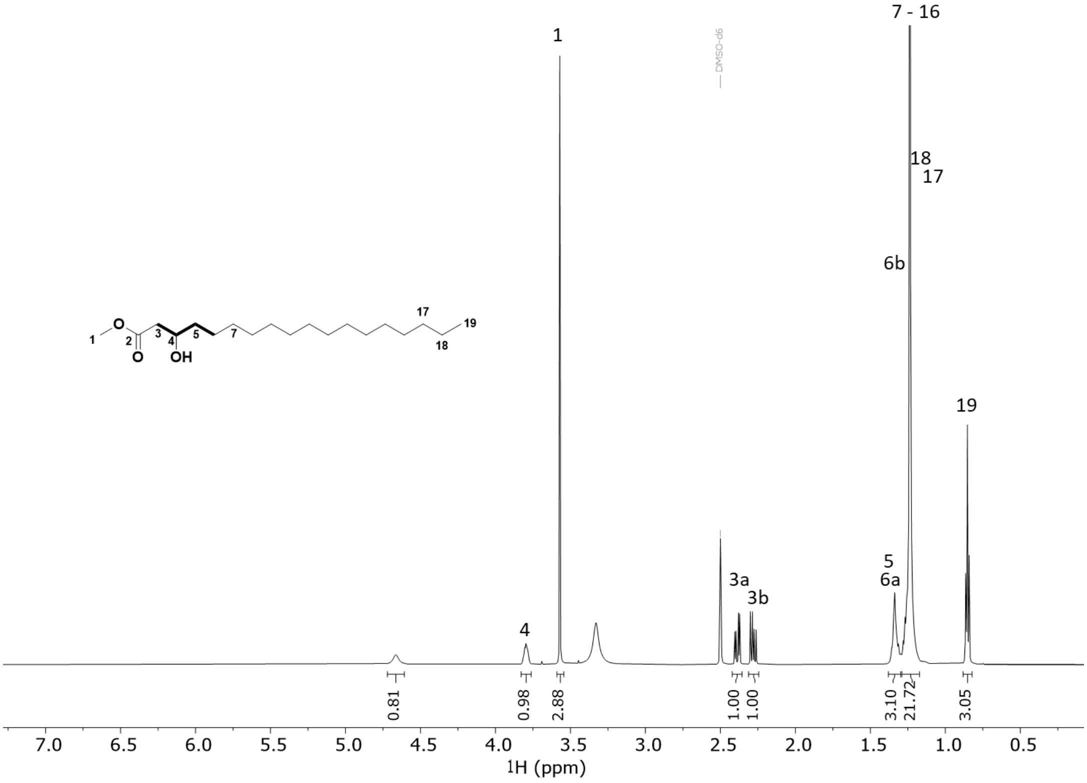

Figure S2. ^{1}H NMR spectrum of Compound D.

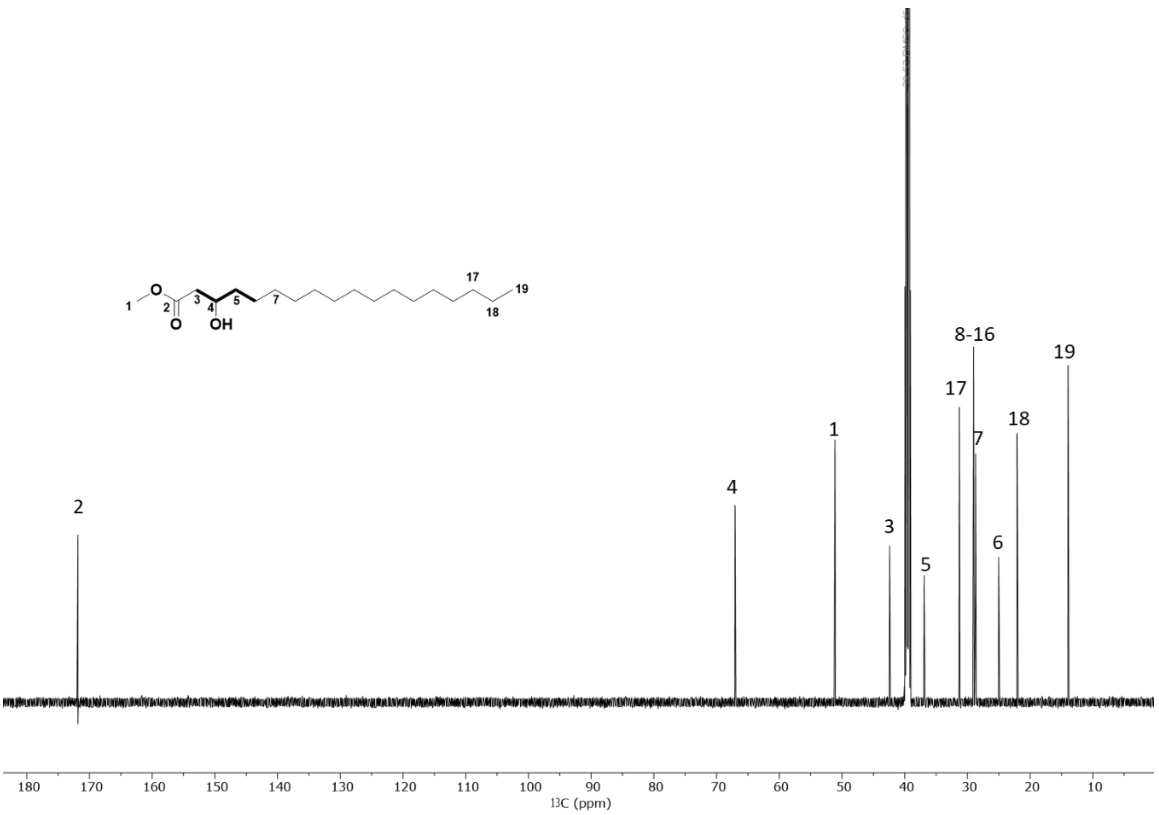

Figure S3. ^{13}C NMR spectrum of Compound D.

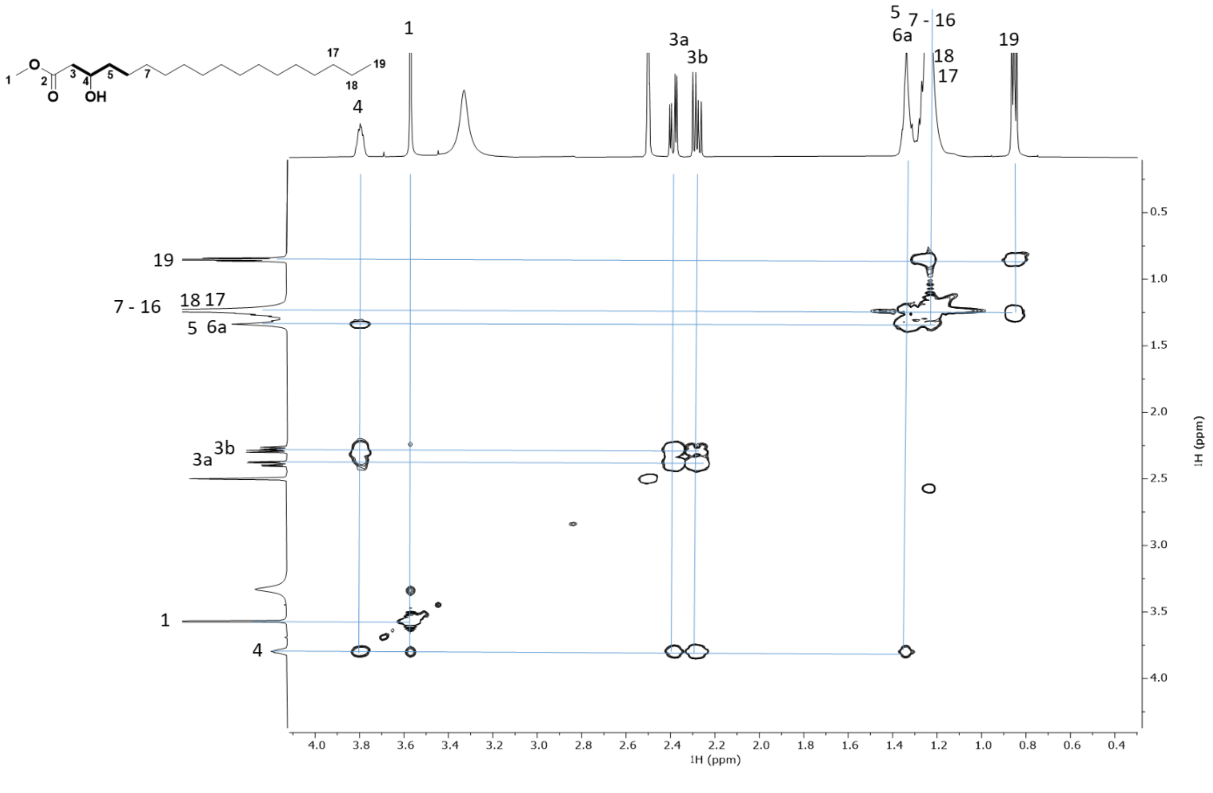

Figure S4. HSQC + HMBC spectrum of Compound D.

S6. Mass Spectrometry Data

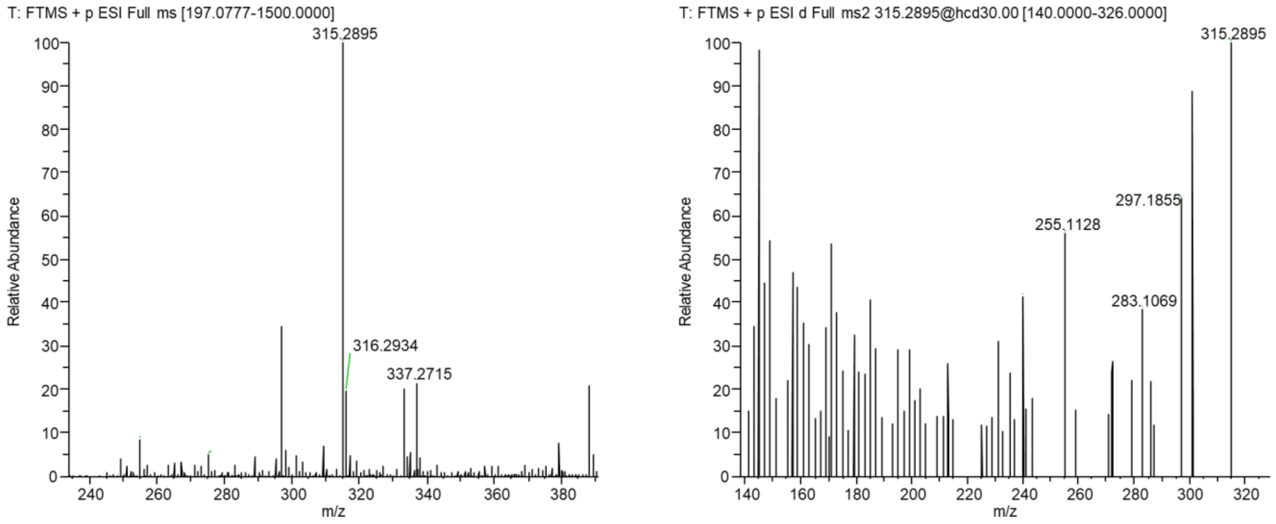

Figure S5: High-resolution mass spectrum of Compound D.

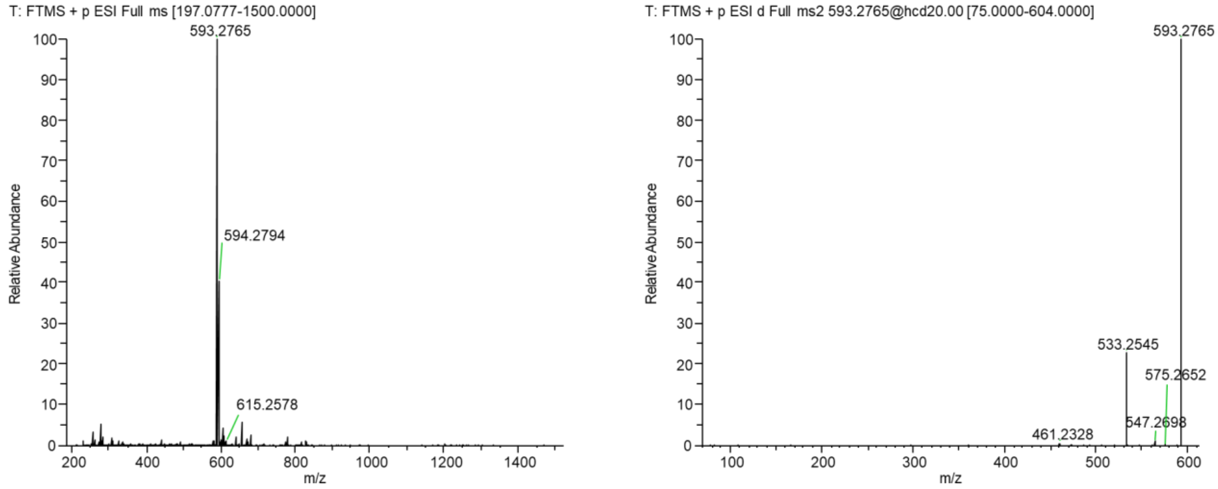

Figure S6: High-resolution mass spectrum of Compound E.
